# GALNT14: An Emerging Marker Capable of Predicting Therapeutic Outcomes in Multiple Cancers

**DOI:** 10.3390/ijms21041491

**Published:** 2020-02-21

**Authors:** Wey-Ran Lin, Chau-Ting Yeh

**Affiliations:** 1 Department of Gastroenterology and Hepatology, Linkou Chang Gung Memorial Hospital, Taoyuan 333, Taiwan; t12360@adm.cgmh.org.tw; 2 Liver Research Center, Linkou Chang Gung Memorial Hospital, Taoyuan 333, Taiwan; 3 College of Medicine, Chang Gung University, Taoyuan 333, Taiwan

**Keywords:** polypeptide *N*-acetylgalactosaminyltransferase, single nucleotide polymorphism, biomarker, cancer

## Abstract

Members of the polypeptide *N*-acetylgalactosaminyltransferase (GALNT) family function as the initiating enzymes that catalyze mucin-type O-glycosylation of proteins, and their dysregulated expression can alter cancer cell behaviors such as de novo occurrence, proliferation, migration, metastasis, and drug resistance. Recent studies have demonstrated that one of the family’s members, GALNT14, is aberrantly expressed in multiple cancers and involved in a variety of biological functions. Moreover, the single nucleotide polymorphisms (SNPs) of *GALNT14*-rs9679162 have been shown to predict therapeutic outcomes in patients with hepatocellular carcinoma as well as several other different types of gastrointestinal cancer. This review summarizes the structural features of GANLT14, its functional roles, and the predictive values of *GALNT14* genotypes and enzyme levels in multiple cancers receiving distinct anticancer therapies.

## 1. Introduction

Glycosylation, an enzymatic process that attaches glycans to proteins or other organic molecules, is essential for multicellular life. Among several types of glycosylation, the addition of a sugar to the oxygen atom of amino acid residues (O-glycosylation) occurs on thousands of secreted and cell surface proteins, altering their structures, functionalities, and subcellular distributions [1,2,3,4]. O-glycans are built by adding sugar molecules to proteins sequentially. The members of the *N*-acetylgalactosaminyltransferase (GALNT) family are enzymes that initiate O-glycosylation by the addition of an *N*-acetylgalactosamine (GalNAc) to a serine or threonine residue of a mucin-type protein [5]. This process plays a pivotal role in the synthesis of Thomsen-nouvelle (Tn) antigens, which are well-characterized tumor-associated molecules [6]. The GALNT families contain 20 members, from GALNT1 to 14 and from GANLTL1 to L6 [5]. In cancer, the alteration of O-glycosylation by GALNTs may affect a variety of biological processes, including tumor progression, proliferation, and migration [7]. 

**Figure 1:**
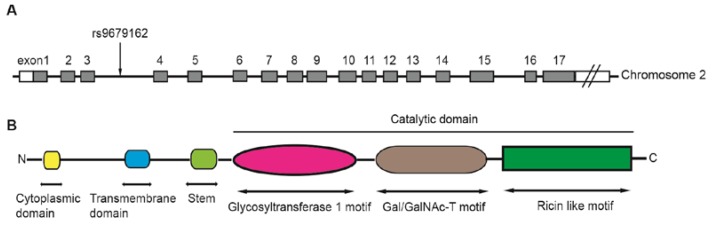
The structure of the *GALNT14* gene, including the location of rs9679162 (**A**), and the common functional domains of GALNT proteins (**B**). The *GALNT14* gene is located on chromosome 2 and includes at least 17 exons. rs9679162 is located between exons 3 and 4 (**A**). The members of the GALNT family possess several common functional domains (**B**). The N-terminal cytoplasmic domain contains 4 to 22 amino acids. The transmembrane domain contains 15 to 25 amino acids. The catalytic region contains more than 450 amino acids, which can be divided into three parts: a glycosyltransferase 1 motif, a Gal/GalNAc-T motif, and a ricin like motif.

*GALNT14* was firstly cloned and identified from the gastric cancer cell line MKN45 in 2003 [8]. The *GALNT14* gene is located at chromosome 2p23.1, spanning over 228 kb and containing at least 17 exons (Figure 1A). It has been found in many human tissues and is highly expressed in the skin and kidneys [8]. 

The GALNT14 protein is a 552-amino acid, type-II membrane protein that consists of an N-terminal cytoplasmic domain, followed by a transmembrane domain, a stem region, and a catalytic domain (Figure 1B) [6]. The catalytic domain includes a glycosyltransferase 1 motif, a Gal/GalNAc transferase motif, and a ricin-like lectin motif, which are commonly observed in GALNT family proteins [9]. The glycosyltransferase 1 motif appears to be responsible for Mn^2+^ coordination and binding both GalNAc and ribose on the sugar nucleotide donor [10]. The Gal/GalNAc transferase motif contains the catalytic general base of the β1,4-galactosyltransferase family. The ricin-like lectin motif contains three homologous repeats (α, β, and γ) and may function to modulate and improve the catalytic efficiency of Gal/GalNAc transferase [5].

The functions of GALNT14 in various cancers have been studied, including the alteration of apoptotic signaling [11], change of tissue invasiveness, and modulation of migration properties [12,13,14,15]. GALNT14 expression also regulates multi-drug resistance in breast cancer cells [16]. Clinically, the GALNT14 level serves as a prognostic marker for neuroblastoma [17] and non-small cell lung cancer (NSCLC) [15]. It has been proposed as a predictive marker for Apo2L/TRAIL-based anticancer therapy [11]. Finally, a single nucleotide polymorphism (SNP) in the *GALNT14* gene, rs9679162, has been identified as a prognostic marker for several gastrointestinal cancers. This review summarizes our current knowledge of GALNT14 functions in various tumors, so as to provide insight into its predictive values in the therapeutic outcomes of cancers.

## 2. Discovery of a GALNT14 SNP, Capable of Predicting Therapeutic Outcomes for Hepatocellular Carcinoma (HCC) Patients Receiving Systemic Chemotherapy, Transcatheter Arterial Chemoembolization (TACE), Hepatic Arterial Infusion Chemotherapy (HAIC), and Sorafenib Treatment

The predictive value of the GALNT14-rs9679162 SNP was first discovered in patients with advanced HCC receiving systemic chemotherapy. Subsequently, this SNP was found to associate with the outcomes of several other anti-HCC treatments, including TACE, HAIC, and sorafenib. These results are summarized in Table 1.

### 2.1. GALNT14-rs9679162 SNP Discovery and Its Association with HCC Under Chemotherapy

The predictive value of the GALNT14-rs9679162 SNP in cancer was first discovered in a study attempting to identify germline SNP markers capable of predicting the treatment responses of advanced HCC patients receiving chemotherapy using 5-fluorouracil, mitoxantrone, and cisplatin (FMP) [18]. Two experimental steps were conducted in this study. Firstly, a genome-wide association study (GWAS) was performed in a cohort of 16 patients who either suffered from rapid disease progression (n = 9) or had partial (n = 5) or complete (n = 2) treatment responses, retrospectively. Among 16 SNPs on chromosomes 2, 6, 15, and X achieving allelic Chi-square test p-values smaller than 0.0001, GALNT14-rs9679162 had the lowest p-value (*p* = 0.000017), as assessed using the kernel-based association test. Secondly, validation of SNP rs9679162 in association with therapeutic responses was performed in an independent cohort of 41 patients prospectively. In this validation cohort, a significant association was found again between rs9679162 and the therapeutic responses (*p* = 0.006326). A follow-up survival analysis demonstrated that patients with the “TT” genotype had better progressive-free survival (PFS) in both retrospective and prospective cohorts (*p* = 0.00041 and 0.01485, respectively) than “non-TT” (“GT” + “GG”) genotypes. However, the overall survival (OS) was only significantly different in the retrospective cohort (*p* = 0.00622), not in the prospective cohort. This study demonstrated firstly that GALNT14-rs9679162 genotypes are potentially associated with the responses of the first course of FMP chemotherapy in patients with advanced HCC and could serve as a predictor marker for HCC. 

To confirm the predictive value of GALNT14-rs9679162, a prospective study was conducted in advanced HCC patients receiving a less toxic, split-dose FMP protocol [19]. One hundred and seven patients with advanced HCC (Bacelona Clinical Liver Cancer stage C with either main portal vein thrombosis and/or distal metastasis) were enrolled and treated by split-dose FMP therapy. Of these patients, 28 (26.2%) had the “TT” and 79 (73.8%) had the “non-TT” genotype. The patients with the “TT” genotype, when compared to “non-TT”, had better prognoses, including longer OS (6.8 vs. 3.9 months, *p* < 0.001) and PFS (3.9 vs. 2.1 months *p* < 0.001), and a better response rate (28.6% vs. 10.1%, *p* = 0.029) and disease control rate (35.7% vs. 15.2%, *p* = 0.030). A further multivariate analysis confirmed that the rs9679162 genotype was an independent predictor for OS (*p* = 0.002). A categorical analysis showed that a subgroup of patients with the “TT” genotype, who had a tumor size <10 cm and neutrophils <74%, had the best outcome (median OS 25.5 months, therapeutic response rate 47.1%). This prospective study confirmed that the GALNT14-rs9679162 SNP is an effective predictor for therapeutic outcomes in advanced HCC patients receiving split-dose FMP chemotherapy.

To evaluate whether a combination of the SNP and other clinical parameters together with on-treatment side effects could serve as an effective predictor for favorable outcomes, 118 advanced HCC patients receiving split-dose FMP were retrospectively enrolled in the study [20]. Results showed that pretreatment with α-fetoprotein (AFP) at a dose of ≤2800 ng/mL (median level), presence of the GALNT14 “TT” genotype, on-treatment leukopenia, and absence of vomiting were independent predictors for favorable PFS (*p* = 0.001, 0.035, 0.008, and 0.009, respectively) and OS (*p* = 0.028, 0.006, 0.027, and 0.013, respectively). A total of 30 patients (25.4%) with both AFP ≤2800 ng/mL and the GLANT14 “TT” genotype exhibited longer median PFS and OS (3.11 vs. 2.11 months, *p* = 0.014; and 5.75 vs. 3.93 months, *p* = 0.001, respectively), and nine patients (7.6%) with all four favorable factors exhibited the longest median PFS and OS (10.64 vs. 2.07 months, *p* = 0.002; and 25.50 vs. 4.50 months, *p* < 0.001, respectively). These results suggest that a lower AFP level in combination with the GALNT14 “TT” genotype could serve as favorable pre-therapeutic predictors for advanced HCC patients receiving FMP chemotherapy.

The distribution of different GALNT14 genotypes in HCC has been demonstrated to be related to HCC etiologies and ethnicities [21]. The “TT” genotype was found to be lower in percentage among patients with virus-originated HCC compared with those with non-viral HCC (22.57% vs. 47.06%, *p* = 0.023). The proportion of the “TT” genotype in Chinese population was shown to range from 24.18% to 30.15% in different cohorts. It was significantly higher in Japanese and African populations (42.11%–54.55%, *p* < 0.0001), but significantly lower in an Italian cohort (7.84%, *p* = 0.004). 

### 2.2. GALNT14-rs9679162 SNP Association with HCC Patients Receving TACE

TACE is currently the standard treatment in HCC patients with Barcelona Clinic Liver Cancer stage B. A cohort of 327 HCC patients treated with TACE was enrolled to investigate the predictive prognosis value of the GALNT14-rs9679162 genotype [22]. Again, the “TT” genotype was associated with better prognosis, including a shorter time-to-response (HR = 2.362, *p* < 0.001) and time-to-complete response (HR = 1.947, *p* = 0.004) and a longer PFS (*p* < 0.001), when compared with the “non-TT” genotype. In patients with albumin <3.5 g/dL, the “TT” genotype was associated with longer OS (*p* = 0.027). This study showed that GALNT14 genotypes were also significantly associated with clinical outcomes of HCC patients receiving TACE.

### 2.3. The Prediction Value of the Combination of GALNT14-rs9679162 and Other SNPs in Advanced HCC Patients Receiving Chemotherapy

GALNT14-rs9679162 has been demonstrated to be capable of predicting chemotherapy responses in advanced HCC [18]. However, the GWAS also identified several other candidate SNP markers, including rs6025211, rs715171 (x-linked), LOC105369482-rs1955024, and WWOX-rs13338697. The prognostic value of these candidate markers was evaluated in an independent cohort of 116 advanced HCC patients receiving split-dose FMP chemotherapy [23]. It was shown that the “TT” genotype of GALNT14 remained an effective predictor for favorable time-to-tumor progression (TTP) and OS (*p* = 0.012 and 0.002). Moreover, the WWOX-rs13338697 “CT” genotype was associated with an unfavorable TTP (*p* = 0.031), and the rs6025211 “CT” genotype was associated with an unfavorable OS (*p* = 0.014). When these three SNPs were combined, patients with GALNT14-rs9679162 “TT”/WWOX-rs13338697 “non-CT” genotypes achieved the most favorable treatment outcomes (n = 19; median TTP, median OS and response rate were 3.9 months, 6.8 months, and 4/19 (21.1%), respectively), whereas patients with GALNT14-rs9679162 “non- TT”/rs6025211 “CT” genotypes were associated with the most unfavorable treatment outcomes (n = 40; median TTP, median OS and response rate were 1.9 months, 3.5 months, and 1/40 (2.5%), respectively). The remaining patients had intermediate clinical outcomes. This study suggests that pretreatment genotyping of these SNPs could help to decide whether these patients should receive chemotherapy or other treatments.

### 2.4. A GALNT14 -rs9679162 Genotype-Guided Therapeutic strategy for Advanced HCC

Although targeted agents are recommended as the first-line treatment for advanced HCC, they were not able to significantly prolong survival in certain advanced HCC patients with distal metastasis. On the other hand, systemic chemotherapy or hepatic arterial infusion chemotherapy (HAIC) could achieve a complete response in a small proportion of patients. In order to identify the subgroups of HCC patients with the best outcomes out of those receiving either chemotherapy, HAIC, or targeted agents, previously identified SNP predictors (GALNT14-rs9679162, WWOX-rs13338697, and rs6025211) were tested in a real-world cohort of 237 advanced HCC patients (171 receiving systemic FMP chemotherapy followed by various anticancer treatments including sorafenib; 66 receiving HAIC) [24]. The results showed that patients with all three SNPs with favorable chemotherapy outcomes (GALNT14-rs9679162 “TT”, WWOX-rs13338697 “non-CT” and rs6025211 “non-CT”) had the best complete response rate and median OS (35.3%, 17.8 vs. 5.3 months, *p* = 0.024), compared with the remaining patients. When the three favorable SNP markers were combined with two clinical criteria (tumor diameter <8 cm, neutrophils <80%), the complete response rate to chemotherapy reached 60%. Subsequent sorafenib for chemotherapy non-responders was associated with longer OS (*p* < 0.01). Surprisingly, the GALNT14 “GG” genotype was associated with longer OS (*p* < 0.001, median OS > 10.5 months) in HAIC treated patients.

To evaluate the predictive role of GALNT14-rs9679162 in patients receiving sorafenib treatment, a cohort of 81 HCC patients treated by sorafenib as the first-line therapy was studied [24]. The results showed that the “TT” genotype was not associated with OS when all patients were included. However, it was found that the “TT” genotype was associated with a longer OS (*p* = 0.027) and the “GG” genotype was associated with a shorter OS (*p* = 0.006) in patients who were positive for the anti-hepatitis C virus antibody. In patients with who were positive for the hepatitis B virus surface antigen, no significant association was found between the genotypes and prognosis (*p* > 0.05 for all comparisons). 

## 3. *GALNT14-rs9679162* SNP Association with Different Gastrointestinal (GI) Cancers

The predictive value of the *GALNT14-*rs9679162 SNP has been studied in various gastrointestinal (GI) cancers, including esophageal squamous cell carcinoma, gastric signet ring cell carcinoma, colorectal adenocarcinoma, pancreatic adenocarcinoma, and cholangiocarcinoma (Table 2).

### 3.1. Esophageal Squamous Cell Carcinoma

Concurrent chemoradiotherapy (CCRT) is the most common treatment for patients with locally advanced, unresected esophageal squamous cell carcinoma (ESCC) to prolong patient survival [25,26]. However, CCRT has a high-toxicity profile which restricts its clinical use. In order to select a suitable patient group for CCRT, the GALNT14-rs9679162 SNP has been examined as a therapeutic response predictor in ESCC [27]. A cohort of 108 patients with ESCC receiving CCRT was recruited. Among these patients, the percentages of patients with GALNT14-rs9679162 “TT”, “TG”, and “GG” genotypes were 28 (25.9%), 51 (47.2%), and 29 (26.9%) respectively. While the genotypes were not associated with the OS, the “GG” genotype was associated with a lower rate of CCRT response (24.1% vs. 50.6%, *p* = 0.014). Further multivariate Cox-proportional hazard model analysis showed that the “GG” genotype was associated with a longer time to complete/partial response (HR = 0.385, *p* = 0.022). Since the presence of a complete/partial response to CCRT was critical for advanced ESCC patients to achieve better outcomes, the “GG” genotype can be used as an unfavorable predictor for CCRT in advanced CCRT. 

### 3.2. Gastric Signet Ring Cell Carcinoma

Gastric signet ring cell carcinoma (SRCC) is one type of gastric cancer carrying a poorer prognosis compared with other types of gastric cancer [28]. However, the prognostic factors for gastric SRCC itself have seldom been delineated. To investigate whether the GALNT14-rs9679162 SNP can be used as a prognostic marker in gastric SRCC, a cohort of 347 patients with gastric SRCC receiving surgical resection was recruited for the study [29]. The percentages of patients with “TT”, “TG”, and “GG” genotypes were 23.34%, 59.65% and 17%, respectively. The results showed that the “TT” genotype was independently associated with an unfavorable OS (HR = 1.550, *p* = 0.048) in advanced stage SRCC patients (TNM stage IIB to IV). The subgroup analysis further revealed that the ”TT” genotype was associated with unfavorable OS in SRCC patients harboring more aggressive phenotypes such as node status > 0 (HR = 1.808, *p* = 0.0013), lymphatic invasion (HR = 1.587, *p* = 0.021), vascular invasion (HR = 3.389, *p* = 0.0076), and perineural invasion (HR = 1.604, *p* = 0.0161). This suggested that gastric SRCC could be stratified into different prognostic subgroups by the combination of clinicopathological factors and the GALNT14 genotype. The poorest prognosis subgroup included patients with the aggressive phenotype together with the GALNT14 “TT” genotype.

### 3.3. Colorectal Adenocarcinoma

Adjuvant oxaliplatin-based chemotherapy has been suggested as a standard treatment for patients with stage III colorectal adenocarcinoma following surgical resection. Although adjuvant chemotherapy has been shown to reduce around 30% of disease recurrence and 22% to 32% of mortality, not all patients benefit from adjuvant therapy [30,31,32]. Therefore, the predictive role of the GALNT14-rs9679162 SNP was investigated in this group of patients [33]. A cohort of 300 patients with stage III colorectal adenocarcinoma receiving curative resection followed by oxaliplatin-based chemotherapy was retrospectively recruited. Of these patients, 18% of patients had the “TT” genotype and harbored an unfavorable OS (HR = 5.406, *p* = 0.019). A subgroup analysis further showed that the “TT” genotype was associated with an unfavorable OS in the following subgroups: age ≤ 65 years, male, left side CRC, N2 stage, carcinoembryonic antigen >5 ng/mL, and mucinous histology (*p* = 0.012, 0.011, 0.009, 0.025, 0.013, and 0.007, respectively). This study concluded that the “TT” genotype is an unfavorable prognostic marker in patients with stage III colorectal adenocarcinoma receiving oxaliplatin-based adjuvant chemotherapy. 

### 3.4. Pancreatic Ductal Adenocarcinoma 

Pancreatic ductal adenocarcinoma (PDA) is one of the most aggressive cancers and is associated with a poor prognosis due to its advanced presentation at diagnosis and limited therapeutic options. The lack of validated predictive markers further complicates this situation [34]. Again, the predictive value of the GALNT14-rs9679162 SNP genotype was examined in PDA patients undergoing surgical resection [35]. A cohort of 103 patients with PDA was enrolled for analysis. The GALNT14 genotype analysis revealed that 19.4%, 60.2%, and 20.4% of patients had the “TT”, “TG”, and “GG” genotypes, respectively. Patients with the “GG” genotype had a longer mean OS time compared with that of those with the “non-GG” genotype (37.1 vs. 16.1 months, *p* = 0.005). Further univariate analysis followed by multivariate Cox proportional hazard analysis identified the “GG” genotype, negative resection margin, and locoregional disease as independent predictors for favorable OS (*p* = 0.003, *p* = 0.037, *p* = 0.021, respectively). The study suggested that the “GG” genotype could serve as a favorable prognostic marker in patients with resected PDA.

### 3.5. Cholangiocarcinoma

Cholangiocarcinoma emerges from the dysregulated proliferation of cholangiocytes and is notorious for its poor prognosis and response to chemotherapy [36]. The association between the prognosis of patients with resected cholangiocarcinoma and the GALNT14-rs9679162 SNP genotype was examined [37]. A cohort of patients with cholangiocarcinoma (n = 112) was retrospectively recruited. Of these patients, 31.3%, 49.1%, and 19.6% had “TT”, “TG”, and “GG” genotypes, respectively. The “TT” genotype was associated with unfavorable OS in the univariate analysis (HR = 2.282, *p* = 0.023). Furthermore, two tumor characteristics, perineural and vascular invasion, were independently associated with unfavorable OS (*p* = 0.001 and *p* = 0.002, respectively). In a multivariate linear analysis, the “TT” genotypes were independently associated with two known predictors of unfavorable prognosis, perineural invasion (*p* = 0.035) and lymph node metastasis (*p* = 0.005). This study concluded that the “TT” genotype was associated with perineural invasion and lymph node metastasis, as well as unfavorable OS in patients with resected cholangiocarcinoma.

## 4. GALNT14 Enzyme Level Is Associated with the *GALNT14* SNP Genotype

Previous studies of the predictive role of the *GALNT14*-rs9679162 SNP showed that in HCC and esophageal cancer (“carcinoma”), patients with the “TT” genotype have a better prognosis, whereas in other cancers (“adenocarcinoma”), patients with the “GG” genotype have better outcomes [19,22,27,29,33,35,37]. Since GALNT14 enzyme levels have been associated with cancer characteristics, it can be speculated that GALNT14 enzyme expression levels are associated with different SNP genotypes. This hypothesis has been examined in HCC [22] and PDA [35] tissues. The protein levels of GALNT14 and cFLIP-S, an inhibitor of the apoptotic signal transduction, in relation to the *GALNT14* genotype were invested in a cohort of 44 patients who had undergone surgical resection of HCC [22]. Analysis of quantitative protein levels in the genotype-stratified patient subgroups showed (i) the cancer parts had higher GALNT14 protein levels than those of the noncancer parts (*p* = 0.008 and < 0.001 for “TT” and “non-TT” genotypes, respectively), and (ii) a significantly higher cancer to noncancer (C/non-C) ratio of GALNT14 expression was noted in the “TT” genotype compared with the “non- TT” genotype (*p* = 0.001). Additionally, the C/non-C ratios of cFLIP-S were significantly lower in the “TT” genotype than those in the “non-TT” genotype (*p* = 0.014). Glycoproteomic analysis showed that the glycosylated residues of death receptor 5 (DR5) in the “TT” patient-derived HCC tissues were clustered in the two major glycosylation sites, in contrast to a scattered glycosylation pattern in the “non-TT” liver tumor tissues. These molecular findings suggest that the cancerous tissues from HCC patients with the “TT” genotype are more sensitive to extrinsic apoptosis signaling compared with those with the “non-TT” genotype. 

In PDA, a preliminary assessment of the GALNT14 enzyme expressions from 20 patients (10 with “TT” and 10 with “GG” genotype) was performed [35]. Among them, patients with the “TT” genotype had higher expression levels of GALNT14 while those with the “GG” genotype exhibited lower GALNT14 levels in the cancerous cells, although the pattern was reversed in the islet cells. This finding suggests that the GALNT14 protein in PDA has an oncogene-like function, similar to what has been reported in breast and ovarian cancers [13,14]. 

## 5. GALNT14 Enzyme Levels Are Associated with the Aggressiveness of Breast and Ovarian Cancers, NSCLC, and Neuroblastoma

### 5.1. Breast Cancer

The association between cancer characteristics and GALNT14 protein expression has been studied in breast cancer [38]. GALNT14 was found to be over-expressed in most breast cancer tissues (47/56, 83.9%), but in only 7/48 (14.6%) of non-malignant breast tissues. A higher histological grade of invasive ductal carcinoma corresponded to a lower expression level of GALNT14. Moreover, Song et al. reported that GALNT14 expression in breast cancer was linked to lung metastasis [13]. High expression of GALNT14 in advanced breast cancer was associated with shorter lung metastasis-free survival (*p* = 0.0015). GALNT14 promotes breast cancer metastasis to the lungs by enhancing the initiation of metastatic colonies and their subsequent growth into overt metastases. The inhibitory effect of lung-derived bone morphogenetic proteins (BMPs) on cancer self-renewal was overcome by GALNT14, which facilitated metastasis initiation within the lung microenvironment. In addition, GALNT14 not only enhanced the recruitment of macrophages to the site of metastases, but also exploited macrophage-derived fibroblast growth factors (FGFs). Moreover, KRAS-PI3K-c-JUN signaling was identified as an upstream pathway that accounted for the elevated expression of GALNT14 in lung-metastatic breast cancer. In a study conducted with the MCF-7 breast cancer cell line, overexpression of GALNT14 significantly enhanced cell proliferation, migration, and tumor invasion, while the knockdown of GALNT14 reduced clonogenicity and attenuated cell migration and cell invasion [12]. Moreover, GALNT14 mediated O-glycosylation of EGF-containing fibulin-like extracellular matrix protein 2 (EFEMP2) which significantly increased the invasion ability of breast cancer cell lines (MCF-7 and MBA-MD-231) [39]. The chemosensitivity of breast cancer was also found to be associated with GALNT14. Osterix decreased chemosensitivity and enhanced anti-apoptosis by upregulating *GALNT14* [40]. Further analysis of 129 breast cancer patients showed that high expression of GALNT14 in breast cancer tissues was associated with higher HER2 (76.7% vs. 58.1%, *p* = 0.038), higher clinical stages (*p* < 0.0001), and shorter DFS (*p* = 0.029). Another study showed that *GALNT14* regulated the stability of P-pg, the efflux pump localized on the cell membrane, which further promoted multidrug resistance [16]. These studies suggested GALNT14 might play an important role in modulating breast cancer aggressiveness and could be considered a therapeutic target for treatment.

### 5.2. Ovarian Cancer

Overexpression of GALNT14 was found in ovarian cancer [14,41]. Knockdown of *GALNT14* significantly suppressed cell migration and cellular morphology change through aberrant glycosylation of transmembrane mucin 13 [14]. Yang et al. demonstrated that the expression levels of miR-125a were downregulated and negatively related to GALNT14 expression in ovarian cancer tissues [42]. Moreover, luciferase reporter assay identified *GALNT14* as a direct target of miR-125a, and overexpression of miR-125a markedly reduced the expression of GALNT14. Both miR-125a mimics and *GALNT14* siRNA suppressed the activity of matrix metalloproteinase (MMP)-2 and MMP-9, further inhibiting extracellular matrix degradation. A recent study showed that aberrant expression of BORIS (Brother of the Regulator of Imprinted Sites) altered cell migration and invasion via upregulation of *GALNT14*, suggesting that BORIS is a potential therapeutic target in ovarian high-grade serous carcinoma [43].

### 5.3. NSCLC

The expression of GALNT14 was highly associated with a shorter RFS in a clinicogenomics study that included 138 NSCLC patients [44]. In PDA, melanoma, and NSCLC cell lines, GALNT14 expression was correlated with Apo2L/TRAIL sensitivity [11]. Overexpression of *GALNT14* increased cellular Apo2L/TRAIL sensitivity, whereas RNA interference of *GALNT14* expression reduced responsiveness. Through the modulation of extracellular DR4 and DR5 O-glycosylation sites, GALNT14 enhanced apoptotic signaling. Immunohistochemistry assays that measured GALNT14 expression have been developed to select NSCLC patients who might be more sensitive to the proapoptotic receptor agonists dulanermin (rhApo2L/TRAIL) and drozitumab (DR5-agonist antibody) [45]. Moreover, GALNT14 was shown to increase the sensitivity of WNT signaling and increase the stability of the β-catenin protein, leading to induced HOXB9 expression and acquisition of an invasive phenotype [15]. A meta-analysis of clinical genomics data showed that overexpression of GALNT14 or HOXB9 was strongly correlated with reduced RFS and increased HR, suggesting that targeting the GALNT14/WNT/HOXB9 axis might be a novel therapeutic approach to inhibit NSCLC metastasis.

### 5.4. Neuroblastoma

A *GALNT14* mutation (c.802C > T) was identified as a neuroblastoma predisposition gene and was predicted to be functionally damaging by the PolyPhen2 (Polymorphism Phenotyping v2) and SIFT (Sorting Intolerant From Tolerant) scoring methods [17]. Furthermore, high expression of GALNT14 was associated with a worse OS in a public dataset of 88 neuroblastoma samples. *GALNT14* is located close to *ALK* on 2p23.1, a region previously discovered to be linked with neuroblastoma.

## 6. Conclusions

GALN14, an initiating enzyme of O-glycosylation, has been demonstrated to play pivotal roles in cancer cell proliferation, migration, and metastases. The expression level of *GALNT14* as well as its SNP genotype can be used to predict clinical outcomes in various types of cancer. However, the relationships between the *GALNT14* SNPs, the enzyme expressions, and the tumor behaviors have not been fully unveiled. Here, we summarized the current knowledge of GALNT14 in various cancers, particularly focusing on its role as a biomarker. Further studies are still needed to provide the missing mechanisms regarding the links between the genotypes and expression levels, as well as other GALNT14-involved microenvironmental elements that can modulate cancer behaviors. For example, HCC is tightly linked to liver cirrhosis and male gender, and thus it will be interested to know whether GALNT14 is involved in modulating fibroblast or sex hormone behavior. Hopefully, with this knowledge, new therapeutic strategies can be devised in the future.

## Figures and Tables

**Table 1:** *GALNT14* SNPs at different HCC stages and treatments.

HCC Stage and Treatment	Case Number	Favorable Genotype (% of Total Patients)	Prognostic Value	Reference
Advanced HCC receiving FMP C/T	16 patients for SNP discovery (retrospective cohort) 41 patients for confirmation (prospective cohort)	“TT” (37.5% in retrospective cohort; 43.9% in prospective cohort)	1. In retrospective cohort, longer PFS (*p* = 0.00041) and OS (*p* = 0.0062) 2. In prospective cohort, longer PFS in prospective cohort (*p* = 0.01485)	[18]
Advanced HCC receiving split-dose FMP C/T	107 patients (prospective cohort)	“TT” (26.2%)	1. Favorable OS (HR = 0.385, *p* = 0.002) 2. Favorable one-year survival rate (32.1% vs. 11.4%, *p* = 0.018) 3. Favorable PFS (HR = 0.431, *p* = 0.001) 4. Favorable CT response (28.6% vs. 10.1%, *p* = 0.029) 5. Favorable disease control rate (35.7% vs. 15.2%, *p* = 0.030)	[19]
Advanced HCC receiving split-dose FMP C/T	118 patients (retrospective cohort)	“TT” (25.4%)	1. Combination of AFP ≤ 2800 ng/mL, longer PFS (3.11 vs. 2.11 months, *p* = 0.014); longer OS (5.75 vs. 3.93 months, *p* = 0.001) 2. Combination of AFP ≤ 2800 ng/mL, leukopenia, absence of vomiting, longest median PFS (10.64 vs. 2.07 months, *p* = 0.002); longest OS (25.5 vs. 4.5 months, *p* < 0.001)	[20]
Advanced HCC receiving TACE	327 patients (retrospective cohort)	“TT” (29.1%)	1. Shorter time-to-response (HR = 2.362, *p* < 0.001) 2. Shorter time-to-complete response (HR = 1.947, *p* = 0.004) 3. Longer PFS (*p* < 0.001) 4. In patients with albumin < 3.5g/dl, longer OS (*p* = 0.027)	[22]
Advanced HCC receiving split-dose FMP C/T	171 patients (retrospective cohort)	GALNT14 “TT”, WWOX-rs13338697 “non-CT” rs6025211” non-CT”(9.9%)	1. Favorable CR rate (35.3%); 2. Longer OS (*p* = 0.024) 3. Combined with tumor diameter <8 cm and neutrophil < 80%, best CR rate (60%) 4. For chemotherapy non-responder, soranefib has longer OS (*p* < 0.01)	[24]
Advanced HCC receiving HAIC	66 patients (retrospective cohort)	“GG” (16.7%)	1. Longer OS (*p* < 0.001)2. Median OS > 10.5 months	[24]
Advanced HCC receiving sorafenib as first-line therapy	81 patients (retrospective cohort)	“TT” in patients with positive anti-HCV	1. Longer OS (*p* = 0.027)	[24]

Abbreviations: AFP, α-fetoprotein; C/T, chemotherapy; HR, hazard ratio; OS, overall survival; PFS, progressive free survival; FMP, 5-fluorouracil, mitoxantrone, cisplatin; SNP, single nucleotide polymorphism; TACE, transcatheter arterial chemoembolization; *GALNT14*, N-acetylgalactosaminyltransferase14; HAIC, hepatic arterial infusion chemotherapy; HCC, hepatocellular carcinoma.

**Table 2:** *GALNT14-rs9679162* SNP in different gastrointestinal (GI) cancers.

Type of GI Cancer	Case Number and Treatment	Unfavorable Genotype (% of Total Patient)	Prognostic Value	Reference
Esophageal squamous cell carcinoma	108 patients with advanced esophageal squamous cell carcinoma with CCRT	“GG” (26.1%)	1. Lower rate of CCRT response (24.1% vs. 50.6%, *p* = 0.014) 2. Longer time to complete/partial response (HR 0.385, *p* = 0.022)	[27]
Gastric signet ring cell carcinoma	347 patients with gastric signet ring cell carcinoma receiving surgical resection	“TT” (23.3%)	1. In advanced stage (IIB to IV) group, unfavorable OS (HR = 1.550, *p* = 0.048) 2. In N > 0 subgroup, unfavorable OS (HR 1.808, *p* = 0.0013) 3. In lymphatic invasion subgroup, unfavorable OS (HR 1.587, *p* = 0.0021) 4. In vascular invasion subgroup, unfavorable OS (HR 3.389, *p* = 0.0076) 5. In perineural invasion subgroup, unfavorable OS (HR 1.604, *p* = 0.0161)	[29]
Colorectal adenocarcinoma	300 patients with stage III colorectal cancer with oxaliplatin based adjuvant CT	“TT” (18.1%)	1. Unfavorable OS (HR = 5.406, *p* = 0.019) 2. In age ≤ 65 y subgroup, unfavorable OS (HR = 5.051, *p* = 0.024) 3. In male subgroup, unfavorable OS (HR = 7.337, *p* = 0.030) 4. In left side colorectal cancer group, unfavorable OS (HR = 7.857, *p* = 0.026) 5. In N2 stage subgroup, unfavorable OS (HR = 6.017, *p* = 0.049) 6. In CEA > 5 ng/mL subgroup, unfavorable OS (HR = 11.295, *p* = 0.049) 7. In mucinous histology subgroup, unfavorable OS (HR = 13.296, *p* = 0.037)	[33]
Pancreatic ductal adenocarcinoma	103 patients with pancreatic adenocarcinoma receiving surgical resection	“Non-GG (TG + TT)”(80.6%)	1. Lower mean OS time (16.1 vs. 37.1 months, *p* = 0.005) 2. Unfavorable OS (HR = 3.663, *p* = 0.003)	[35]
Cholangiocarcinoma	112 patients with cholangiocarcinoma receiving surgical resection	“TT” (31.3%)	1. Unfavorable OS (HR = 2.282, *p* = 0.023) 2. Associated with perineural invasion (*p* = 0.035) 3. Associated with LN metastasis (*p* = 0.005)	[37]

Abbreviations: CCRT, concurrent chemoradiotherapy; HR, hazard ratio; OS, overall survival; CEA, carcinoembryonic antigen; LN, lymph node; C/T, chemotherapy; *GALNT14, N*-acetylgalactosaminyltransferase14.

## Abbreviations

MDPI	Multidisciplinary Digital Publishing Institute
DOAJ	Directory of open access journals
TLA	Three letter acronym
LD	Linear dichroism
